# The Mevalonate Pathway, a Metabolic Target in Cancer Therapy

**DOI:** 10.3389/fonc.2021.626971

**Published:** 2021-02-25

**Authors:** Borja Guerra, Carlota Recio, Haidée Aranda-Tavío, Miguel Guerra-Rodríguez, José M. García-Castellano, Leandro Fernández-Pérez

**Affiliations:** Molecular and Translational Pharmacology Lab, Institute for Biomedical and Health Research (IUIBS), University of Las Palmas de Gran Canaria, Las Palmas de Gran Canaria, Spain

**Keywords:** mevalonate, cholesterol, oxysterols, isoprenoids, sterol regulatory element binding protein, cancer, statins

## Abstract

A hallmark of cancer cells includes a metabolic reprograming that provides energy, the essential building blocks, and signaling required to maintain survival, rapid growth, metastasis, and drug resistance of many cancers. The influence of tumor microenviroment on cancer cells also results an essential driving force for cancer progression and drug resistance. Lipid-related enzymes, lipid-derived metabolites and/or signaling pathways linked to critical regulators of lipid metabolism can influence gene expression and chromatin remodeling, cellular differentiation, stress response pathways, or tumor microenviroment, and, collectively, drive tumor development. Reprograming of lipid metabolism includes a deregulated activity of mevalonate (MVA)/cholesterol biosynthetic pathway in specific cancer cells which, in comparison with normal cell counterparts, are dependent of the continuous availability of MVA/cholesterol-derived metabolites (i.e., sterols and non-sterol intermediates) for tumor development. Accordingly, there are increasing amount of data, from preclinical and epidemiological studies, that support an inverse association between the use of statins, potent inhibitors of MVA biosynthetic pathway, and mortality rate in specific cancers (e.g., colon, prostate, liver, breast, hematological malignances). In contrast, despite the tolerance and therapeutic efficacy shown by statins in cardiovascular disease, cancer treatment demands the use of relatively high doses of single statins for a prolonged period, thereby limiting this therapeutic strategy due to adverse effects. Clinically relevant, synergistic effects of tolerable doses of statins with conventional chemotherapy might enhance efficacy with lower doses of each drug and, probably, reduce adverse effects and resistance. In spite of that, clinical trials to identify combinatory therapies that improve therapeutic window are still a challenge. In the present review, we revisit molecular evidences showing that deregulated activity of MVA biosynthetic pathway has an essential role in oncogenesis and drug resistance, and the potential use of MVA pathway inhibitors to improve therapeutic window in cancer.

## Introduction

Adaptive metabolic reprogramming is often observed in cancer cells. It is widely accepted that metabolic disruptions of carbohydrates, proteins, and lipids are one of the hallmarks of cancer (1–3). Metabolic adaptations provide energy and the crucial building blocks needed to maintain abnormal survival, rapid growth, metastasis, and drug resistance in many tumors. In addition to tumor microenviroment, they are main driving forces for cancer progression (4, 5). Lipid metabolism reprograming involves lipid-related enzymes, metabolites, and signaling pathways linked to key regulators that can directly influence gene expression and chromatin remodeling, cellular differentiation, stress response pathways, or tumor microenviroment that collectively drive tumor development (6). An elevated or deregulated activity of mevalonate (MVA)/cholesterol biosynthetic pathway in specific cancer cells suggests that they are dependent of the continuous availability of MVA-derived metabolites (7–10). Furthermore, the aberrant activity of 3-hydroxy-3-methylglutaryl coenzyme A (HMGCoA) reductase (HMGCR), the rate-limiting enzyme of MVA pathway, can promote malignant transformation (7) and provides essential metabolites (i.e., sterols and non-sterol intermediates) that collectively drive tumor growth and development. Despite clinical evidences supporting the use of MVA pathway inhibitors (i.e., statins) for limiting cancer morbimortality are relatively low, increasing preclinical (11–19) and epidemiological (20–28) studies sustain the inverse association between statins and cancer-specific mortality rate. This beneficial effects of statins have been described in several types of cancer, including osteosarsocoma/chondrosarcoma (16–18), prostate (24, 26), colon (29, 30), breast (19, 31), liver (32, 33), pancreas (34), ovarian (35, 36), esophageal (37, 38), lung (39), and hematological malignances (40). Interestingly, statins may suppress epithelial-mesenchymal transition (EMT) program together with the inhibition of cancer stem cell generation, maintenance, and expansion (6, 41). Unluckily, the use of statins in cancer is currently limited by the requirement of using high doses for prolonged periods, thus generating adverse effects. Therefore, studies focused on elucidating new strategies targeting the MVA signaling pathway to improve the therapeutic window in cancer are urgently needed (42). Clinically relevant, synergistic effects of tolerable doses of statins with conventional chemotherapy could enhance treatment efficacy, by reducing doses of each drug and, probably, adverse effects. To date, clinical trials that identify combinatory therapies (statins-chemotherapy) that improve therapeutic window in different cancer types are still a challenge. In this review, we revisit preclinical and molecular evidences showing that aberrant MVA biosynthetic pathway may has an essential role in oncogenesis and we discuss how potent inhibitors of MVA pathway may best be applied to improve cancer therapy.

## The MVA Biosynthetic Pathway

**Figure 1:**
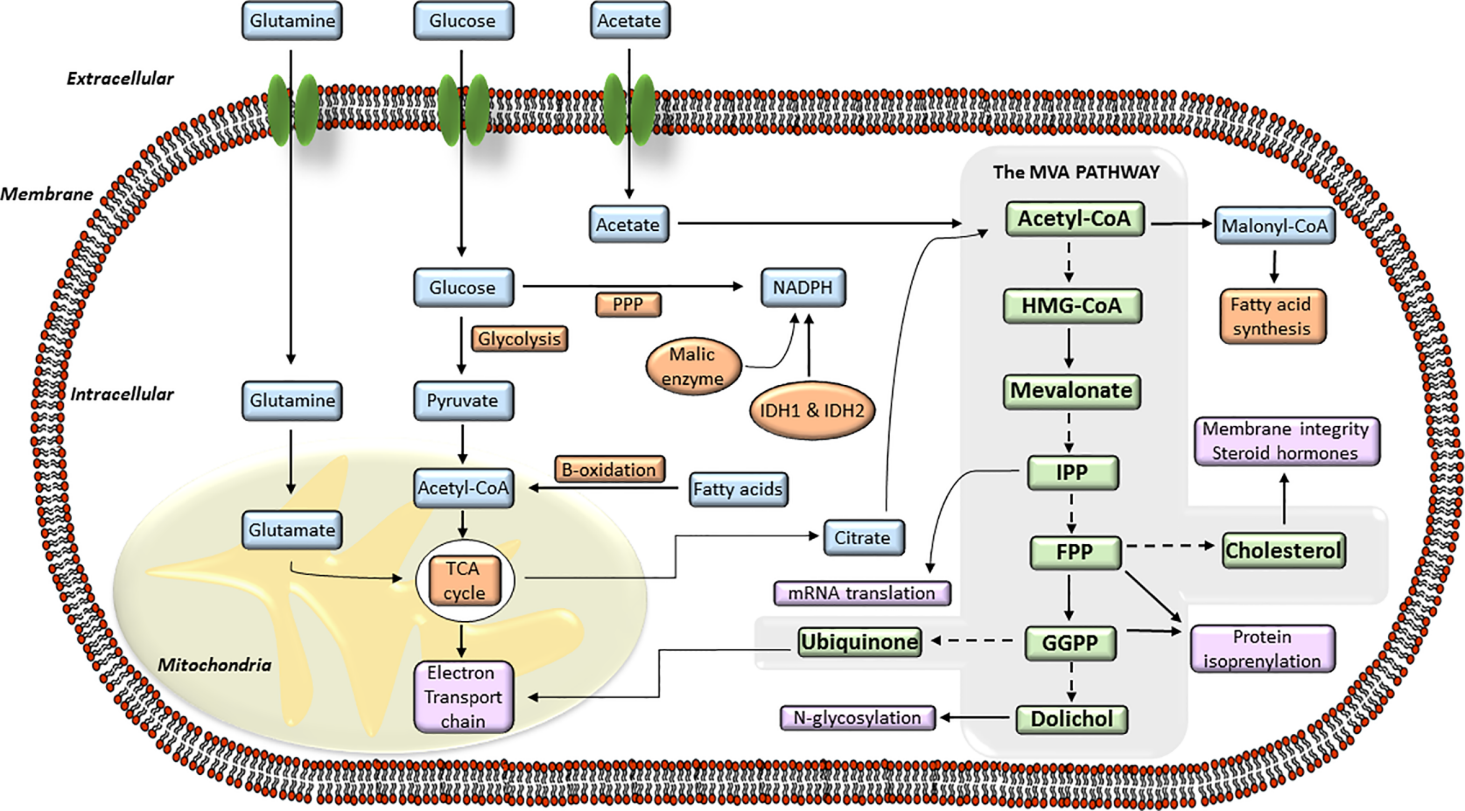
The mevalonate (MVA) pathway and its connection with the intracellular energy metabolism signaling. Diagram of the different steps of the intracellular MVA anabolic pathway, from the entry of acetyl-coenzyme A (CoA) to the production of isoprenoid metabolites. Acetyl-CoA is transformed into hydroxy-methylglutaryl-CoA (HMG-CoA) which is used by the enzyme hydroxy-methyl-glutaryl-CoA reductase (HMGCR) to synthesize MVA. MVA is further metabolized to farnesyl pyrophosphate (FPP), a precursor of cholesterol and sterols. FPP is also converted to geranylgeranyl pyrophosphate (GGPP), and these lipids are used for post-translational modification of proteins, including N-glycosylation and protein prenylation.

In normal cells, cellular cholesterol can arise from receptor-mediated uptake of LDL-cholesterol from circulation, or be *de novo* synthesized from acetyl-CoA by the MVA biosynthetic pathway. The precise regulation of MVA pathway is essential to guarantee continuous production of MVA-derived products, and to guard cells from accumulation of toxic end products, including cholesterol (43, 44). MVA pathway produces lipoproteins, dolichol, ubiquinone or cholesterol derived products (i.e., steroid hormones, oxysterols, vitamin D, bile acids) which are essential regulators of cellular metabolism. Cholesterol is essential for the buildup and maintenance of the structure and function of cellular membranes, cholesterol-rich microdomains or membrane rafts (lipid rafts). These structures constitute a core of organization for several signaling pathways and intracellular transport systems where cholesterol acts as a signaling molecule. The MVA biosynthetic pathway (**Figure 1**) starts with the formation of HMGCoA from three molecules of acetyl-CoA (43, 45), the end product of glycolysis. This reaction is catalyzed by the enzyme HMGCoA synthase. Subsequently, HMGCR converts HMGCoA to MVA which is the rate-limiting step of whole MVA pathway. The MVA is phosphorylated by the MVA kinase and converted to isopentenyl pyrophosphate (IPP). This step is decisive for the biosynthesis of farnesyl pyrophosphate (FPP) and geranylgeranyl pyrophosphate (GGPP) and is regulated by a cascade of different synthases including the farnesyl diphosphate synthase (FDPS) and the GGPP synthase (GGPS). Then, FPP can be converted to squalene and, subsequently, by further enzymes such as squalene synthase and squalene epoxidase, to cholesterol. Further lipid products FPP downstream include dolichol and ubiquinone, both with antioxidant properties, and crucial for glycosylation and mitochondrial electron transport processes. The synthesis of FPP and GGPP is essential for protein prenylation, a key posttranslational modification for localization, membrane anchoring and function of many signaling proteins. Protein prenylation is mediated by the enzymes farnesyltransferase (FTase) I and geranylgeranyl transferases (GGTase) I and II. The MVA pathway also participates in other biological mechanisms including long-term memory of innate immune cells, survival, and polarization of effector immune cells (i.e., macrophages) or metabolic reprograming in cancer cells (46–48).

## Regulation of the MVA Biosynthetic Pathway

The MVA biosynthetic pathway is regulated by transcriptional and post-transcriptional mechanisms including modulation of gene transcription, mRNA translation, protein degradation, and enzymatic activity (44, 49). The HMGCR enzyme, which regulates the rates of cholesterol synthesis, is in turn controlled by very fine-tuned regulatory mechanisms. Transcriptional regulation of HMGCR is mediated by two members of the sterol regulatory element binding proteins (SREBP) family called SREBP1 and SREBP2 (44, 49). SREBP proteins are encoded by two separate genes, SREBP-1 and -2. An alternative splicing of SREBP-1 can be produced, driving the synthesis of two isoforms, SREBP-1a and -1c. Whereas SREBP-1 has been clearly associated with homeostasis of cholesterol and fatty acids, SREBP-2 is mainly involved in synthesis and uptake of cholesterol. Thus, in response to intracellular sterol levels, SREBPs regulate the MVA biosynthetic pathway. Briefly, when the amount of intracellular sterol increases, SREBPs are held in an inactive form at the endoplasmic reticulum (ER) by their binding partner SREBP cleavage-activating protein (SCAP) and the insulin-induced genes (INSIG)-1 and -2. However, in response to sterol deprivation (e.g., when HMGCR activity is inhibited), intracellular end products of the MVA biosynthetic pathway are depleted. As the number of sterols diminishes, they no longer bind SCAP, thus producing a conformational change that triggers the SCAP-SREBP complex dissociation from the INSIGs and translocation from the ER to the Golgi. The SREBPs are successively cleaved by Golgi-resident proteases and released on their activated form, so they can translocate to the nucleus where they bind to sterol regulatory elements (SRE). This initiates the transcription of target genes that translate into key proteins involved in the biosynthesis of MVA-derived metabolites (i.e., HMGCoA synthase, HMGCR, FPP synthase, Insig-1) and cholesterol uptake (i.e., LDLR) to restore intracellular isoprenoid and sterol levels. Intracellular sterol levels are also regulated by oxysterols, metabolites derived from cholesterol oxidation. The 7α- and 27-hydroxycholesterols are synthesized in the liver by CYP7A1 and CYP27A1, the genes encoding the rate-limiting enzymes of neutral and acid bile synthetic pathways, respectively, which contribute to eliminate cholesterol. Oxysterols contribute to cholesterol homeostasis through activation of Liver X receptors (LXR) (50). LXR were originally characterized by their role in the positive regulation of the gene CYP7A. This relevant physiological role was further confirmed by the phenotype of LXRα null mice, which appear healthy when fed on a standard mouse diet but, when fed with a cholesterol-enriched diet, failed to induce CYP7A (51). Consequently, LXRα null mice suffered from a dramatic accumulation of cholesteryl ester in the liver and a reduction in bile acid production. Upon binding to LXR, oxysterols induce the transcription of specific ATP-binding cassette (ABC) transporters A1 and G1, that increase cholesterol efflux from enterocytes and macrophages, respectively (52). In addition to LXR activation, oxysterols (e.g., 25-hydroxysterol (25HC)), and high sterol concentrations, lanosterol, or Insig, can provoke ubiquitination and proteasomal degradation of HMGCR (53). Furthermore, the sterol-accelerated degradation of HMGCR is strengthen by non-sterol isoprenoids, including derivatives from FPP and GGPP. Notably, lanosterol does not interact with the sterol-sensing domain of SCAP and, therefore, does not suppress the processing of SREBP. Thereby, oxysterols downregulate HMGCR by increasing its ubiquitination-mediated degradation as well as suppress HMGCR gene transcription by inhibiting the delivery of SREBP-SCAP complex from ER. In contrast, lanosterol enhances the HMGCR degradation rate, and cholesterol limits the translocation of SREBP-SCAP (54). Furthermore, negative feedback responses of IPP, FPP, and GGPP suppress the activity of the MVA kinase. Expression of HMGCR is further modulated at the translational level, where the translation rate of HMGCR mRNA is controlled by the demand of the cell for non-steroid isoprenoids (e.g., MVA). When HMGCR dependent-MVA production is inhibited by statins, HMGCR mRNA is efficiently translated, even in the presence of sterols, being in contrast reduced when MVA is added. Finally, as mentioned below, the catalytic activity of HMGCR can be inhibited *via* phosphorylation by AMPK, a sensor of cellular energy state (55).

## The MVA Pathway in Cancer

**Figure 2:**
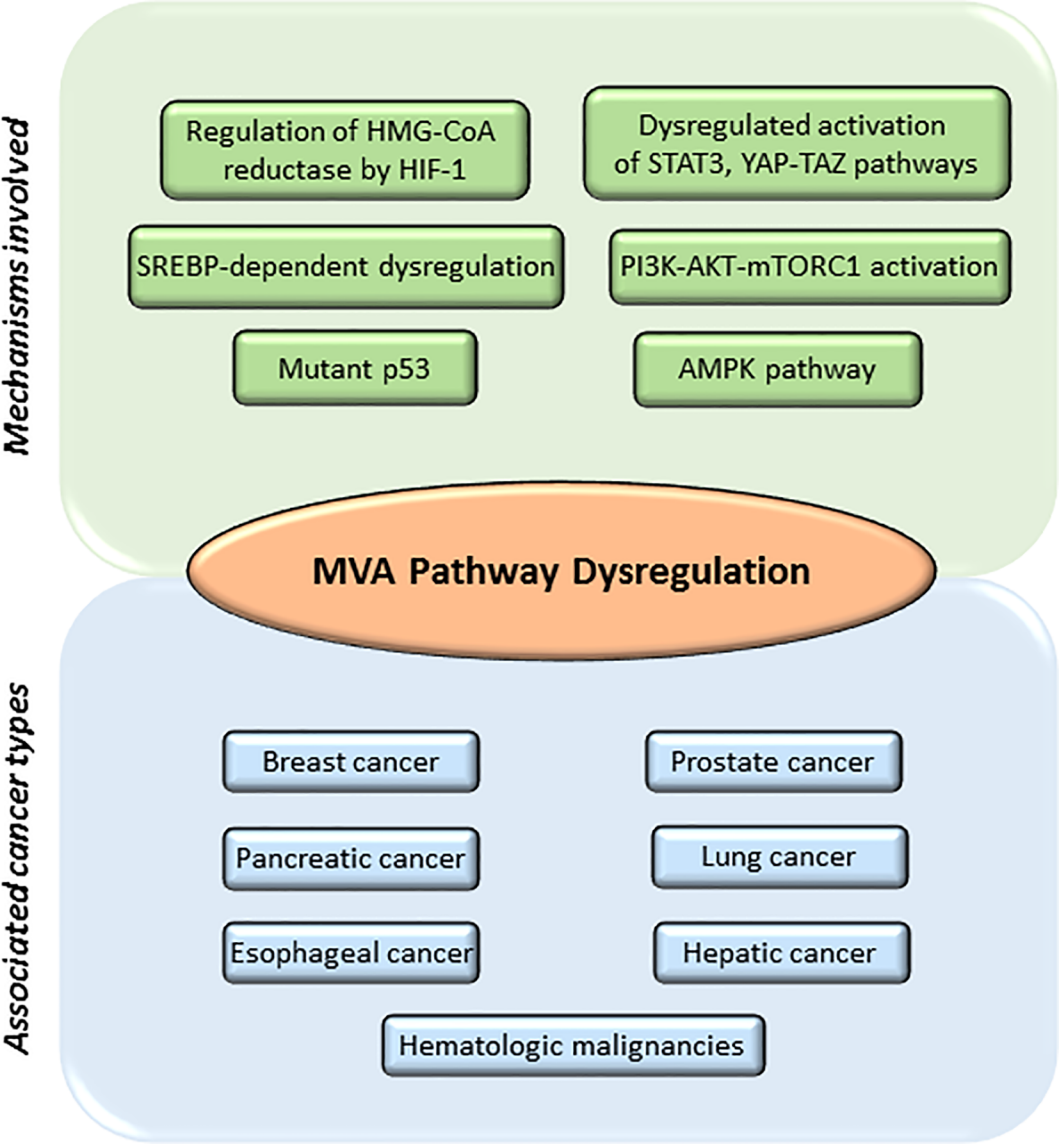
Main mechanisms involved in mevalonate (MVA) pathway dysregulation and different cancers associated. MVA pathway is upregulated in several cancers including breast, prostate, pancreatic, lung, esophageal, hepatic, and leukemia. Main mechanisms involved in the dysregulation of MVA pathway include: abnormal regulation of the enzyme hydroxy-methyl-glutaryl-CoA reductase (HMGCR) by different transcription factors such as hypoxia-inducible factor 1 (HIF-1); mutations or abnormal activation of sterol regulatory element-binding proteins (SREBPs); mutations on tumor suppressor proteins such as tumor protein (p53); decreased AMP-activated protein kinase (AMPK) activation; increased activation of signaling pathways such as phosphoinositide 3-kinase (PI3K)—protein kinase B (AKT)—mammalian target of rapamycin complex 1 (mTORC1), Janus Kinase (JAK)/Signal Transducer and Activator of Transcription 3 (STAT3) or Hippo signaling pathway (YAP-TAZ).

The Warburg phenomenon (56) is the best studied metabolic adaptation program developed by cancer cells. It was described as the preference of cancer cells to use aerobic glycolysis to obtain most of their energy, even in the presence of abundant oxygen supply, when normal cells would typically use the aerobic cellular respiration. Therefore, tumor cells will be highly dependent on glucose to produce large quantities of energy and provide other cells with intermediates necessary for the biosynthesis of amino acids, nucleic acids, and lipids (5, 57). This metabolic reprograming provides energy, the crucial building blocks, and signaling required to keep survival, rapid growth, and drug resistance of many cancers (1–3). Glycolysis generates acetyl-CoA (45), a molecule that is derived from acetate and/or glutamine metabolism. Acetyl-CoA can be incorporated into the MVA biosynthetic pathway, into lipids by fatty acid synthase (FAS) or into phospholipids by the action of different enzymes, including the pro-oncogene choline kinase (CK) (58). Acetyl-CoA feeds the MVA biosynthetic pathway to generate metabolites that are essential to maintain survival and rapid growth of multiple tumors. Accordingly, transcriptional profiling studies support the hypothesis that genes involved in cholesterol and fatty acid metabolism are upregulated in cancer cells and play an essential role in transformation (59). Several studies suggest that an elevated requirement for cholesterol is an innate metabolic hallmark in cancer cells, which could be used in a prophylactic and therapeutic manner. However, the complexities of how lipid metabolism interconnects with oncogenesis and tumor progression are not yet well understood. The list of molecules functionally connected with the MVA biosynthetic pathway in cancer is wide and diverse. It includes: 1) enzymes [e.g., HMGCR (8, 60–62), small GTPases (63, 64), ATP citrate lyase (ACL) (5), AMPK (65–67), FAS (68), pyruvate kinase M2 (PKM2) (69)]; 2) CD36, a fatty acid transporter (68); 3) signaling pathways [e.g., PI3K-AKT-mTOR (70, 71), Hippo (72, 73), Hedgehog (74, 75)]; 4) transcriptional regulators [e.g., SREBPs (68, 76), HIF-1 (77), STAT3 (78–80)], c-MYC (6, 81), YAP/TAZ (72, 73); and 5) nuclear receptors such as LXRs (82), ERα (62, 83), and Estrogen-Related Receptor (ERRα) (84, 85). Additionally, the loss-of-function of tumor suppressor proteins such as p53 (86, 87) and pRb (88–90) can also contribute to adapt lipid metabolism to tumor growth, metastasis, and drug resistance (**Figure 2**).

## HMGCR

Originally, the hypothesis that MVA-derived metabolites have a role in cancer cell biology was suggested by studying liver cancer (91) and primary chronic lymphocytic leukemia cells (92). Further gene expression profiling and inmunohystochemical analysis identified that HMGCR expression can be associated with a molecular gene signature of certain subtypes of breast cancer (93). The proto-oncogenic role of HMGCR was functionally shown by overexpression of constitutively active HMGCR, which potentiated both anchorage-independent cellular growth in soft agar as well as the development of xenografts (7). Furthermore, dysregulated HMGCR was shown to induce anchorage-independent growth of an immortalized, non-transformed cell line, and support the formation of myeloid colonies from normal hematopoietic progenitors. A link between MVA pathway and oncogenic signaling was also reported with the cooperation between HMGCR and the small GTPase Ras to promote cell transformation (7). Clinically relevant, increased levels of HMGCR were shown to correlate with poor prognosis in breast (93) and prostate cancer (94) patients. Several epidemiological studies have also evidenced that hypercholesterolemia and increased oxysterol production are associated with higher cancer risk (e.g., postmenopausal breast cancer, colon cancer, lung cancer, non-Hodking lymphoma, acute mieloide leukemia) (95). Accordingly, high levels of cholesterol could provide cancer cells with immune surveillance and/or resistance to drug therapy (9, 60). Thus, cholesterol is recognized as an inherent metabolic demand in cancer cells and increased rates of cholesterol synthesis can potentiate the progression of numerous types of cancer (7, 8). This is explained by the fact that highly proliferative cancer cells need to rapidly produce membranes, so requiring higher cholesterol availability than normal cells (8). Besides, cholesterol is an integral component of lipid rafts, which constitute a core of organization for several signaling pathways and intracellular transport systems (96), and is also a precursor of downstream products such as oxysterols and steroid hormones which can drive activation of nuclear receptors in several cancers (97). Thus, decreasing intracellular cholesterol biosynthesis could be a promising strategy to restrain cancer progression. Indeed, it was reported that acute myeloid leukemia (AML) cells exposed to high-cholesterol media *in vitro*, increased their cholesterol synthesis and influx compared to their normal, non-tumorigenic counterparts. Moreover, AML cells did not usually display efficient feedback repression of cholesterol synthesis and influx, and this appeared to be associated with increased survival of leukemic cells. Interestingly, synthetic LXR ligands can block tumor cell proliferation, tumorigenesis, and metastasis in multiple cancer models, which emphasizes the potential role of LXRs in cancer therapy (82, 98). Cholesterol is also the precursor of steroid hormones, responsible for driving the initiation and progression of hormone-dependent breast and prostate cancers. Recently, it has been shown that long-term E2 withdrawal of ERα-positive breast cancers triggers to the stable epigenetic activation of the MVA pathway and cholesterol synthesis (61). The resulting augmented level of 27-hidroxycholesterol was enough to induce ERα signaling in the absence of exogenous E2, promoting the activation of genes that give rise to an invasive phenotype (62). Likewise, in prostate cancer, the *de novo* biosynthesis of androgens from cholesterol activates androgen receptor (AR) activity in castration resistant disease (99), thus suggesting a role for the MVA pathway in prostate cancer progression, also considering the observations that SREBP expression is enhanced in advanced stages of prostate cancer. However, these findings require further research into the utility of inhibitors of the MVA pathway and/or SREBPs in the treatment of hormone-driven cancers. All these evidences indicate that increased cellular cholesterol and/or oxysterols, represent another hallmark in many cancers, and suggest that limiting cellular cholesterol levels, or LXR activity, should be considered to improve therapeutic window and sensitivity of cancer cells to chemotherapy. Unluckily, the mechanisms by which HMGCR and the MVA pathway become dysregulated, or how precisely this deregulation promotes carcinogenesis, are still poorly understood, so further studies would be needed in order to elucidate these key questions.

## Small GTPases

Rho GTPases belong to the Ras superfamily which comprises more than 20 members classified into eight subfamilies (Rho, Rac, Cdc42, RhoD/RhoF, RhoH, RhoU/RhoV, Rnd, and RhoBTB) according to their structure and function (63, 64). Most Rho family proteins act as molecular switches cycling between an inactive guanosine diphosphate GDP-bound state in the cytoplasm, and an active guanosine triphosphate (GTP)-bound state in the cell membrane. The activation state of Rho GTPases is tightly regulated and occurs in a cell-type and pathway-dependent manner. Although Rho GTPases are mostly known as master regulators of the actin cytoskeleton, they are also involved in cell proliferation, apoptosis, cell cycle progression, and genomic stability, and they are dysregulated in several human cancers (100). Notably, some Rho GTPases have been related to tumor metabolism through activation of glutaminase, which catalyzes the conversion of glutamine to glutamate and ammonia, a crucial step in glutamine metabolism and contributor to the Warburg phenomenon. As Rho GTPases need isoprenylation to properly function, their activity essentially depends on the HMGCR enzyme, thus providing a critical link between the MVA pathway and tumor cell metabolism. Specifically, the isoprenoids FPP and GGPP post-translationally modify proteins with C-terminal CAAX, CXC or CC motifs, such as small GTPases, with very well established roles in carcinogenesis (100). Rho can only be geranylgeranylated, whereas H-Ras is purely farnesylated, and K-Ras and N-Ras can be both farnesylated and geranylgeranylated. Accordingly, inhibition of the MVA pathway can reduce the isoprenylation of these GTPasas and promote apoptosis of cancer cells (100–102). This antitumoral effect can be prevented by the addition of GGPP, and sometimes FPP, suggesting that these MVA-derived metabolites are vital for cancer cell viability. Isoprenoids are also involved in the production of the ubiquinone (quinone coenzyme Q). In this case, the hydrophobic isoprenoid chain places the ubiquinone to the inner membrane of the mitochondria, where the quinone group transfers electrons from complex I or II to complex III of the electron transport chain (ETC) (103). Therefore, ubiquinone is essential for ATP production in cancer cells that rely on oxidative phosphorylation to generate energy. It seems that the depletion of isoprenoid pools, which potentially affect the many proteins that are isoprenylated, mediates the anticancer activity of HMGCR inhibitors (i.e., statins). However, despite dependency of isoprenoids, inhibitors that directly inhibit isoprenylation of small GTPases have not been a successful anticancer strategy to date because, in part, their narrow therapeutic window.

## CD36

The fatty acid transporter CD36 is considered as a novel connection between lipids and cancer. It may contribute to regulate cholesterol synthesis (88) and phenotypic changes linked to tumor growth and metastasis (68, 104, 105). In hepatocytes, activation of CD36 increases phosphorylation of Ser872 in HMGCR, and the recruitment of the Insig 1/2 contribute to degradation of HMGCR by the ubiquitin-proteasome pathway. In addition, genes encoding key enzymes involved in MVA pathway, and under the control of SREBP2, remained unresponsive to sterol depletion, due to retention of Scap by Insig-1/2. Interestingly, some fatty acids (e.g., palmitic acid), or a high-fat diet, enhance the metastatic potential of cells in a CD36-dependent manner, whereas blocking CD36 causes inhibition of metastasis in mouse models of human oral cancer, with no side effects (104). Relevant to the connection between oncogenic STAT3 and aberrant lipid metabolism, it has been shown that STAT3-activated CD36 contributes to fatty acid uptake in chronic lymphocytic leukemia cells (106), which supports a critical role of STAT3 in the regulation of CD36-dependent leukemia.

## AMPK

The AMP-activated protein kinase (AMPK) was originally described as a protein to lessen anabolic pathways activity when intracellular ATP levels are low (66). AMPK acts as an energy sensor and central regulator of glucose, lipid, and cholesterol metabolism in specialized tissues (e.g., liver, muscle, adipose). This function has placed AMPK as a key therapeutic target in cancer. Decreased AMPK activation can promote carcinogenesis, and the pharmacological induction of AMPK has been reported to be cytotoxic to cancer cells (65, 67). This is in part, because AMPK can regulate the MVA pathway through phosphorylation and thereby inhibition of HMGCR (55) and SREBPs (107) activities. AMPK can phosphorylate the Ser872 within the catalytic domain of HMGCR, suppressing its enzymatic activity, independently of its feedback regulation by MVA-derived metabolites. Moreover, the transcription factors SREBPs are direct targets of AMPK phosphorylation, as AMPK inhibits the proteolytic processing, nuclear translocation, and transactivation activity of SREBPs, after their activation (e.g., under hyperglycemic and hyperinsulinemic conditions). Interestingly, activation of AMPK in the liver of insulin-resistant mice was shown to inhibit the transcription of enzymes that participate in lipid and cholesterol biosynthesis, including HMGCS1 and HMGCR, thereby reducing hepatic triglyceride and cholesterol levels. Thus, AMPK can inhibit the activity of MVA pathway both, directly, *via* HMGCR phosphorylation and, indirectly, through SREBPs inhibition. However, the relevance of this regulation in the context of cancer is still scarcely regarded. The MVA pathway may besides modulate AMPK activity, thereby forming a feedback loop (108). The discovery that the serine-threonine kinase Liver Kinase B1 (LKB1), a known tumor suppressor, was a crucial upstream activator of the AMPK, added a relevant piece of information to our understanding about the connection between cell metabolism and cancer (109). It is therefore feasible that the anticancer effects of AMPK activation and the tumor suppressor role of its upstream kinase LKB, are in part due to the inhibition of HMGCR and the MVA pathway. LKB1 is modified by protein farnesylation and it phosphorylates and activates AMPK. This suggests a negative feedback loop, where AMPK activation, in response to reduced cellular energy, results in the suppression of the MVA pathway *via* the phosphorylation of HMGCR and SREBPs. This reduces in turn the FPP pool inside the cell, thereby hampering LKB1 farnesylation and blocking activation of AMPK. Remarkably, AMPK activation has also been reported to suppress cell proliferation in normal and cancer cells by regulating cell cycle progression or inhibiting protein synthesis (110, 111). In line with this, recent studies have shown that simvastatin, a potent HMGCR inhibitor, induces apoptosis and cell cycle arrest by activating AMPK and inhibiting the Signal Transducer and Activator of Transcription 3 (STAT3) axis, both in liver cancer cells and tumor xenografts (112, 113). However, restoration of MVA reversed the activation of AMPK and the suppression of STAT3 caused by statin treatment. These findings contributed to demonstrate that AMPK induction and STAT3 inhibition in liver cancer cells are dependent on HMGCR activity. Thereby, MVA signaling pathway, AMPK and STAT3 activities may represent potential therapeutic targets in liver cancer.

## The PI3K-AKT-mTORC1 Axis

In normal cells, the mTORC1 activity can be activated by diverse stimuli (i.e., growth factors, nutrients, energy, and stress signals), and key signaling pathways (i.e., PI3K-AKT, MAPK and AMPK), in order to regulate cell growth, proliferation and survival. Upon stimuli, PI3K produces PtdIns(3,4,5)P3 which binds to AKT and 3-phosphoinositide-dependent protein kinase (PDK1). In contrast, the inactivation of AKT is regulated by PTEN that converts PtdIns(3,4,5)P3 into PtdIns(4,5)P2, driving to a lower recruitment of AKT to the cell membrane (114, 115). An increased mTORC1 activity is observed in 40–90% of the most frequent human cancers. The aberrant activation of PI3K-AKT-mTORC1 signaling leads to an increase activity of the MVA biosynthetic pathway and lipogenesis, and the reprograming of lipid metabolism contributes to potentiate tumor growth (70, 71). Several mechanisms are implicated, including the inactivating mutation of PTEN (116, 117), the mutation/amplification of PI3K-AKT (118), the hyperactivation of PI3K-AKT signaling pathway by growth factors (e.g., insulin, PDGF, VEGF, HER-2, IGF-I), the overexpression of mTORC1 targets (i.e., S6K1, 4BP1, eIF4E), or the loss of tumor suppressors (e.g., PTEN, LKB1, or TSC). These are common mechanisms that increase *de novo* cholesterol synthesis and fatty acid biosynthesis in cancer (119, 120). Upregulated PI3K-AKT activity increases glucose uptake and glycolysis rate in cancer cells, a mechanism that provides NADPH and acetyl-CoA to the MVA pathway. Conversely, inhibition of the MVA pathway can decrease PI3K activity. The PI3K-AKT-mTOR pathway connects with SREBP-mediated lipid biosynthesis by using complex protein-protein interactions and phosphorylation of regulatory elements (121, 122). Interestingly, AKT prevents proteasomal degradation of nuclear SREBPs which increases *de novo* cholesterol and fatty acid biosynthesis. This role of AKT on lipogenesis, and tumorogenesis, is blocked after gene silencing of SREBPs. Furthermore, the connection of mTOR with SREBPs was evidenced by enhanced lipogenesis in response to mTORC1 activation whereas inhibition of mTORC1 with rapamycin blocked both active SREBP and expression of SREBP target genes. In addition to a positive regulation of SREBP, mTORC1 has a main role in regulation of protein synthesis through phosphorylation of downstream effectors such as 4EBP1 and S6K1. Targets of S6K1 include 40S ribosomal protein S6, protein elongation factors, and IGF-II. Clinically relevant, human primary breast cancer samples with high levels of pS6K1, as a marker of mTORC1 activity, had high expression of SREBP target genes (e.g. FASN, LDLR, MVA kinase). In contrast, breast cancer cell lines with silenced SREBPs (1 and/or 2) showed reduced proliferation and increased cell death despite activation of mTORC1. Finally, mTORC1 activation has also been linked to proteins such as STAT3, STAT5 and PPARγ, in a rapamycin sensitive manner. Thereby, aberrant activation of PI3K-AKT-mTOR axis can reprogram protein and lipid biosynthesis in an orchestrated manner to provide efficient tumor growth.

## The Hippo Pathway

The Yes-associated protein (YAP) and the transcriptional co-activator with PDZ-binding motif (TAZ) are key downstream terminal effectors of the Hippo signaling pathway (123). In normal tissues, YAP-TAZ proteins are phosphorylated at specific serine residues in order to confine their subsequent degradation in the cytoplasm (124). However, in cancer, YAP-TAZ proteins are translocated into the nucleus where they bind to TEA domain (TEAD) proteins which drive the transcriptional activation of proliferative genes, the repression of pro-apoptotic genes and the amplification of stem/progenitor cells. Increasing evidences have shown that deregulated Hippo pathway is significantly associated with cancer development (72, 73). Remarkably, YAP and TAZ require the MVA biosynthetic pathway to translocate into the nucleus and be fully functional (72). In fact, it has been reported that the concurrent knockdown of SREBPs (1 and 2) reduces nuclear localization of YAP-TAZ, suggesting the importance of SREBP-mediated induction of the MVA for YAP and TAZ nuclear localization (72). Interestingly, activation of both the MVA pathway and YAP-TAZ is correlated with mutant p53 expression in primary tumors, suggesting a dysfunctional mutant p53-SREBP-YAP-TAZ axis in cancer (72). Relevant to this review, the MVA pathway is an essential intermediate in the oncogenic activation of YAP and TAZ by mutant p53 (72). When statins are used to inhibit the HMGCR activity in the MVA pathway, the nuclear localization and transcriptional activity of YAP-TAZ are also inhibited. GGPP may be involved in this process, as it is known to promote YAP-TAZ nuclear translocation and increase their transcriptional activity *via* activation of Rho GTPases. Thus, when MVA pathway is inhibited, also GGPP is, thereby reducing YAP-TAZ activity. Additionally, it has been shown that YAP-TAZ can be activated by SREBPs, main regulators of MVA pathway, in a breast cancer cell line. Interestingly, mutant p53 promoted YAP-TAZ transcriptional activity and contributed to cancer cell malignancy by maintaining SREBP expression in MVA pathway. Taken together, these data clearly show that MVA participates in the regulation of YAP-TAZ expression and transcriptional activity and reveal an original process through which statins have anticancer effects.

## The Hedgehog Pathway

Members of the Hedgehog (Hh) family of secreted signaling proteins have an essential role in the regulation of vertebrate development and adult tissue homeostasis, including regulation of stem cell physiology (74, 75). Reduced Hh pathway activity can cause development defects in mice and humans, and aberrant increased activity of this pathway is linked to tumorigenesis. The core components of the Hh pathway include: the secreted signaling ligand Hh, the twelve-pass transmembrane receptor Patched (PTCH), the seven-pass transmembrane co-receptor G-protein-coupled receptor (GPCR)-like transducer Smoothened (SMO), and the glioma associated-oncogene (GLI) (74, 75). After secreted from the producing cells, Hh binds to PTCH on the cell surface, and subsequently release suppression of PTCH on SMO. Then, activation of SMO triggers GLI-dependent expression of downstream target genes through a complex network of post-translational modifications and translocations. There are positive and negative feedback loops that ensure a homeostatic regulation of Hh signaling pathway, which include an increment of GLI levels or the potentiation of the activity of negative regulators such as PTCH1, respectively. Relevant to this review, the Hh signaling pathway is regulated by cholesterol and oxysterols, main products of MVA biosynthetic pathway (74). It has been established that cellular cholesterol is an endogenous ligand of SMO. Thereby, cholesterol levels can modulate the Hh signaling pathway by direct binding to GPCR-SMO (50). Thus, cholesterol itself can be used as a substrate for the post-translational modification of Hh ligands, required for biological activities of Hh, as well as a molecule for long-distance and local Hh signal communication. Thereby, inhibitors of MVA pathway (e.g., statins) that modulate Hh pathway activity could represent potential drugs in Hh pathway-related cancers.

## Hypoxia-Inducible Factors (HIF)

Under hypoxic conditions, cells respond by suppressing energy-consuming processes to preserve energy, including mitochondrial respiration (125, 126). These conditions promote the activation of the Hypoxia-Inducible Factors (HIF). The HIF protein family consists of three α members (i.e., HIF-1α, HIF-2α, and HIF-3α) and two β members (i.e., HIF-β and ARNT2), which have a similar domain structure (127). Under hypoxic conditions, HIF-1α is stabilized, binds DNA, and regulates the transcription of glycolytic target genes in cancer cells (125, 128). Several observations have shown that the MVA pathway can be directly or indirectly modulated under hypoxic conditions, in part, because HMGCR expression is regulated through the transcriptional activity of HIF-1α (129, 130). It has been reported that HIF-1α connects pathways for oxygen sensing and feedback regulation of cholesterol synthesis in human fibroblasts by directly inducing the transcription of the INSIG-2 gene. INSIG-2 is an ER membrane protein that inhibits cholesterol synthesis by mediating sterol-induced ubiquitination and subsequent degradation of the HMGCR. Furthermore, pharmacologic stabilization of HIF-1α in the liver was shown to trigger accelerated HMGCR degradation by prior ubiquitination (131). Pharmacologically relevant, in other pathologic fields such as Alzheimer´s disease, statins (simvastatin) have been shown to reduce intracellular levels of HIF-1 expression (132). Likewise, fluvastatin was shown to accelerate ubiquitin/proteasome-dependent degradation of HIF-1, effect that was reversed by concomitant treatment with mevalonate, farnesyl pyrophosphate, or geranylgeranyl pyrophosphate (133). While HIF has been broadly studied as an essential protein for modulation of transcriptional program during the hypoxia response, many other transcription factors (e.g., NFkB, Nrf2, Myc, STAT3) and/or tumor suppressors also function under hypoxic conditions to promote the acquisition and maintenance of metabolic reprogramming phenotype in cancer. Further understanding about the connections between these transcription factors and the MVA pathway constitutes an ongoing challenge.

## Signal Transducers and Activators of Transcription (STAT)

The STAT family of transcription factors consists of 7 members within STAT3 highlights by its oncogenic activity. STAT3 appears constitutively active in a broad variety of cancers that often become addicted to its activity (78–80). In contrast to normal STAT3 activity, which is transient, constitutively active STAT3 is associated with abnormal cell growth and survival, angiogenesis and metastasis, tumor immune evasion, and aberrant mitochondrial function. While tyrosine phosphorylation by Janus Kinases (JAK), represents the main activation mechanism of STATs, alternative mechanisms, such as the interaction with HIF signaling pathway, phosphorylation of STAT3 on S727 (134), and regulation of activity and nuclear traffic by small GTPases (135), appear to play important roles that connect STAT3 oncogenic activities with deregulated metabolism in cancer cells. An important component of STAT3 oncogenic activity resides in the induction of aerobic glycolysis, making cancer cells highly sensitive to glucose deprivation, whereas they are protected from apoptosis and senescence. Accordingly, inhibition of STAT3 tyrosine phosphorylation in several cancer cells down-regulates glycolysis prior to leading to growth arrest and cell death. STAT3-addicted cancer cells can develop a switch towards aerobic glycolysis program through two mechanisms: a) the up-regulation of HIF-1α, which in turn mediates the induction of several glycolytic genes [e.g., hexokinase 2, LDH-A, pyruvate dehydrogenase kinase 1 (PDH), PKM2] (136); and b) the down-regulation of mitochondrial activity, which is totally or partially independent of HIF-1α. HIF-1α induces PKM2 expression, which maintains STAT3 tyrosine phosphorylation, a mechanism that initiates a positive feedback loop that leads breast cancer cells to adapt and grow into hypoxia conditions (137). Similarly, hypoxia can activate oncogenic STAT3 in prostate cancers cells, and, together with the AKT and HIF-pathways, induces an androgen-independent and invasive phenotype (138). In addition, STAT3 phosphorylation on S727 has emerged as a crucial regulator of metabolic processes in the mitochondria. Indeed, S727-STAT3 was found to enhance Complex I and II activities and reduce ROS production within the mitochondria (139, 140). This function appears to be essential for cellular survival under certain stress conditions such as heart ischemia, where mitochondrial STAT3 protects cardiac cells from apoptosis (140). Furthermore, mitochondrial STAT3 potentiates RAS-mediated oncogenic transformation. This finding supports the role of STAT3 in maintaining cell survival and oncogenesis, linked to a metabolic adaptation of cancer cells. In contrast, mitochondrial expression of an inactive mutant S727A-STAT3 was shown to inhibit growth and metastatic capacity of the breast cancer cell line 4T1, and this inhibition correlated with reduction of Complex I activity under hypoxia (141). The form S727-STAT3 can also be induced by the mTOR pathway to potentiate the expression of STAT3 target genes (e.g., Bcl-xL, VEGF, cyclin D2) (142, 143). Moreover, activated forms of small GTPasas such as Rac1, Cdc42 or RhoA directly or indirectly promote the phosphorylation and activation of STAT3 (134, 144, 145). Particularly, Rac1 specifically induces an increase in Rac1 and Cdc42 protein levels and activities, and stimulates autocrine IL6 secretion, which contributes to an increase of STAT3 activity. Interestingly, the activated form of Rac1, but not its inactive variant, forms a complex with STAT3 to regulate its phosphorylation and activity (146, 147). Interestingly, the Rac (and Cdc42) GTPase activating protein MgcRacGAP plays also a critical role in STAT3 activation (148, 149). When activated, the complex MgcRacGAP-Rac-GTP interacts with STAT3 to promote its binding to the IL6 receptor thus facilitating that JAK phosphorylates and activates STAT3. These observations suggest that MgcRacGAP, a core regulator of cytokinesis, and other Rho proteins, support oncogenic properties of STAT3. All observations indicate that STAT3 can integrate different pro-survival and growth signals in a context of energy and respiratory metabolism, emerging as a key molecule to target within the mitochondrial metabolism.

Tumor immune microenvironment (TIM), including surrounding (niche) and inflammatory cells, plays a key role in the development of angiogenesis, proliferation, immunosuppression, and tumor progression (150). These biological effects depend, at least in part, on the aberrant activation of STAT pathway which is an immuno-inflammatory-carcinogenic pathway. Thus, in addition to their roles in adaptive metabolism of cancer cells, aberrant STAT activity can drive cancer development through the regulation of TIM. This is particularly relevant for STAT3 and STAT5 which are highly expressed in Tumor-Associated Macrophages (TAMs), a critical cellular component of TIM. It is well known that TAMs are recruited into tumor formation by chemo-attractant cytokines and, once inside the tumor, tumor cells secrete cytokines that prolong the survival of TAMs; these cells, in turn, express multiple factors that promote tumor development and immunosuppression. STAT3 and STAT5 have been reported to act by inhibiting the antitumor immune response by activating, at least in part, the production of inflammatory cytokines (IL-1, IL-17, IL-10, TGF-β, or VEGF) and promoting tumor growth and metastasis (150). Moreover, it has been described that TAMs could favor the development of tumor resistance to conventional chemotherapy, highlighting the importance of the microenvironment in tumor development. In addition to TAMs, the influence of niche stem cells on tumor development and drug resistant is also relevant. Interestingly, many studies have shown that STAT activity is essential to localize, maintain, and renew Hematopoietic Stem/Progenitors cells (HSPC) into tumoral niche and that STAT hyperactivation is associated with uncontrolled proliferation of HSPC (151–155). Thereby, dual strategies targeting both tumor cell proliferation and tumor niche and/or regulation of TIM represent a promising therapeutic strategy (156, 157). Interestingly, the effects of MVA biosynthetic pathway inhibitors (i.e., statins, bisphosphonates) on TAMs suggest that TIM can be regulated by MVA biosynthetic pathway (158–160). However, despite TIM is known to be highly dependent of cholesterol biosynthesis (10, 161–164), its interplay with MVA biosynthetic pathway and STAT signaling, remains unexplored.

## MYC

MYC belongs to the Myc gene family that is comprised by C-MYC, N-MYC, and L-MYC, and they have been shown to influence almost all aspects of carcinogenesis, including rapid cell growth, inhibition of cell differentiation, genomic instability, metastasis, or angiogenesis (165–167). Aberrant regulation of MYC is observed in more than 50% of cancers, where this oncoprotein is overexpressed, either due to enhanced transcription of the Myc gene or to dysregulated stability of MYC protein. The stability of MYC can be modulated by a) the ubiquitin/26S proteasome pathway, and b) the sequential phosphorylation of MYC at S62 and T58. The phosphorylation of S62 is controlled by the MAPK/ERK pathway and leads to the stabilization of MYC, whereas its phosphorylation on T58 is mediated by GSK3β and promotes ubiquitin-dependent MYC degradation once S62 is dephosphorylated by, for example, the serine/threonine-protein phosphatase 2A (PP2A) (165, 168). Mutations on the phosphorylation sites that stabilize MYC have been identified in human cancers, thus highlighting the relevance of S62 and T58 phosphorylation as regulators of MYC tumorigenic activity (169). MYC is a major driver of metabolic reprogramming in cancer, where this transcription factor regulates the expression of genes involved in anabolic metabolism, cellular bioenergetics and lipid metabolism (167, 170, 171). This oncoprotein can reprogram cancer cell metabolism toward glycolysis and MVA pathway to drive the proliferation and survival of cancer cells. Accordingly, it has been reported that knockdown of c-Myc in gastric cancer cells suppresses glycolysis rates and cell proliferation capacity. MYC can also bind SREBP to drive somatic cell reprogramming into induced pluripotent stem cells (171), or bind to promoters of MVA pathway genes in close proximity to SREBPs (8), suggesting that MYC may contribute to the expression of MVA pathway enzymes. Notably, HMGCR is a positive regulator of phosphorylation, activation, and tumorigenic properties of MYC in a MYC-driven model of hepatocellular carcinoma where exogenous mevalonate deliver can enhance cancer growth (168). In agreement with the positive role of MVA pathway in MYC-induced oncogenic activities, the antitumoral effects of statin were prevented by mevalonate. This effect was associated with a reduction of small GTPase RAC isoprenylation levels and PP2A activation. Moreover, when tumors that expressed active phosphorylated mutants of MYC at S62 or Th58 were studied, there was an increase of tumor resistance to statin treatment which supported the role of serine/threonine phosphatase PP2A as a negative regulator of MYC (168). Recently, studies on MYC null mice showed that mice had improved lifespan, which was linked to the decreased expression of MVA pathway genes, including HMGCR and SREBP2, and most likely to caloric restriction (172). Finally, RAS, whose activity is also regulated by the MVA pathway is thought to modulate MYC activity and enhance levels of HIF-1, independently of hypoxia conditions (173, 174). These findings reinforce the hypotheses that MYC dependent oncogenesis is linked to a deregulated MVA biosynthetic pathway.

## The ERRα Pathway

Estrogen-Related Receptors (ERRs) are a group of nuclear receptors with three isoforms (α, β, and γ) (84, 85, 175). ERRα is mainly expressed in high-energy demanding tissues where it associates with the co-regulator peroxisome proliferator-activated receptor—γ co-activator 1 (PGC-1). In differentiated cells, ERRα, together with PGC, controls cellular metabolism, assists the growth of rapidly proliferating cells, directs metabolic programs necessary for cell differentiation, and keeps cellular energy homeostasis. In several cancer cells, the expression, and the activity of ERRα, and its cofactor PGC-1, is further influenced by oncogenic signals (e.g., IGF1 receptor pathway, estrogen signaling, mTOR pathway) and induces metabolic programs favoring cell growth and tumor progression. This is particularly relevant when there is a functional relation between augmented cholesterol levels and certain cancer phenotypes, with an overexpression of ERRα [i.e. colorectal cancer (176), prostatic, and breast cancers (177)]. Notably, ERRα activity promotes an inflammatory environment by the production of cytokines that supports a protumoral microenviroment (178). Recently, affinity chromatography and transcriptional assays have identified cholesterol as an endogenous ligand and agonist of ERRα (84). A functional link between cholesterol (or MVA pathway) and ERRα has been described in bone, muscle, and in the immune system (macrophages). Particularly, cholesterol-induced bone loss or bisphosphonate osteoprotection are lost in ERRα knockout mice. In addition, statin induction of muscle toxicity and cholesterol suppression of macrophage cytokine secretion are impaired by loss or inhibition of ERRα. These findings showed that cholesterol is an ERRα agonist and that the MVA biosynthetic pathway impacts biological functions of ERRα (85). Thereby, the use of therapeutic strategies that aim to decrease cholesterol levels (e.g., statins, biphosphonates) could be an encouraging way to counteract metabolic reprograming in cancer cells where ERRα plays a critical role.

## ERα

The MVA biosynthesis pathway was recently reported to be up‐regulated in ERα‐positive breast cancer cells lines that are resistant to E2 withdrawal (61, 62, 83). This suggests that dysregulation of cholesterol biosynthesis may be a mechanism of anti-estrogen resistance in ER‐positive breast cancer. Mechanistically relevant, chronic estrogen removal in ERα‐positive breast cancer cells seems to stabilize the epigenetic activation of the MVA pathway and cholesterol biosynthesis (61). This leads to the accumulation of cholesterol-derivative metabolites (i.e., 27HC) which, in the absence of estrogens, acts as ERα agonist, and then potentiates ERα signaling to induce the transcription of genes involved in proliferation and invasion. Therefore, statins might act as anti-breast cancer drug by reducing circulating cholesterol and 27HC, and the availability of these ERα agonists in breast cancer cells. Furthermore, direct suppression of HMGCR by statins depletes intratumoral levels of isoprenoids, which are also key modulators of breast cancer cell proliferation and metastasis.

## Tumor Suppressor Proteins

**Figure 3:**
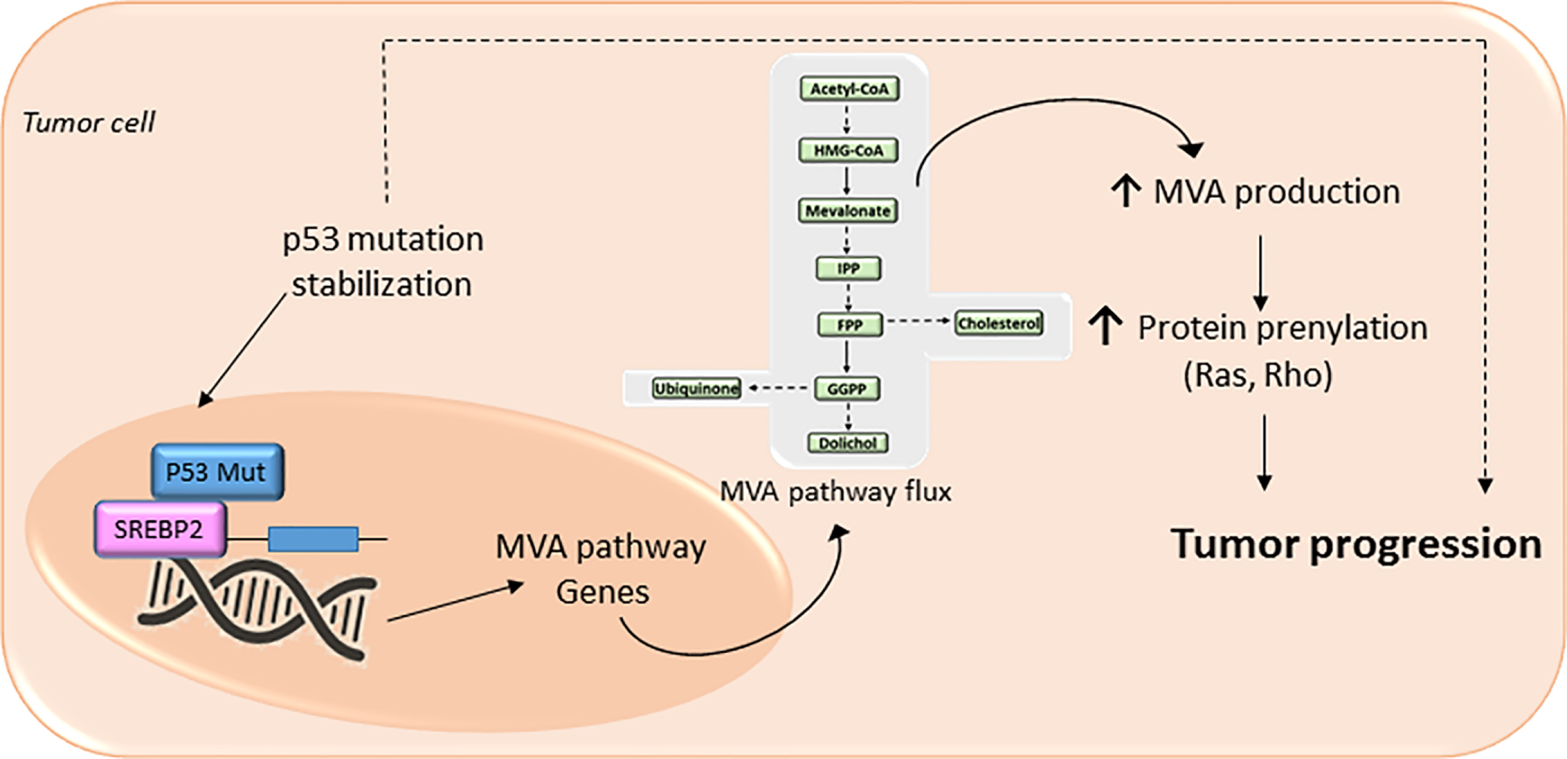
The mevalonate (MVA) pathway in cancer progression. The MVA pathway is dysregulated in several cancer cells due to mutations or abnormal signaling of different proteins/pathways. Upregulation of MVA pathway drives to increased protein prenylation thus promoting a malignant phenotype of cancer cells with an uncontrolled cell invasive growth and survival. In cancer cells expressing a mutation of tumor protein p53, there is a positive-feedback loop where p53 interacts with sterol regulatory element-binding protein (SREBP), leading to increased activation of the MVA pathway activity, and therefore higher levels of MVA. This MVA leads to the stabilization of p53 mutation as well as promotes protein prenylation, thus accelerating cancer progression.

Loss-of-function of tumor suppressor protein p53 (TP53) (86, 87) and cyclin-dependent kinases (cdks)- retinoblastome suppressor protein (Rb)- transcription factor E2F Transcription Factor 1 (E2F1) pathway (88–90), novel regulators of metabolism, promotes the acquisition and maintenance of glucose and/or lipid metabolism reprogramming phenotype in cancer. The mutated forms of the tumor suppressor protein TP53 confer oncogenic properties to p53 in a broad range of cancer types (87). Specific oncogenic mutations lead p53 to functionally interact with nuclear SREBP2 and enhance the transcription of MVA genes (86) (**Figure 3**). Furthermore, an increased expression of mutant p53 in primary breast cancer tissues has been associated to the augmented expression of MVA pathway genes. In contrast, wild-type p53 can decrease lipid synthesis under glucose starving conditions by inducing the expression of phosphatide phosphatase LPIN1, a protein that can prevent SREBPs-DNA binding. Thus, the interaction between p53 and the MVA axis suggests that this pathway may be a novel therapeutic target for tumors with specific p53 gain-of-function mutations. Another example of mutated tumor suppressor gene that leads to an oncogenic phenotype is the cdks-Rb-E2F1 pathway. Analysis of genetically engineered mice deficient in cdk, E2F1, or Rb protein, showed an adaptive reprogramming to metabolism of glucose and/or lipids, including MVA biosynthetic pathway. This showed that the cdk-Rb-E2F1 pathway acts as a key regulator of cell growth, proliferation, and development by sensing external signals that require a particular adaptive metabolic reprograming. Particularly, this cell cycle regulatory pathway is an essential regulator for decreasing oxidative metabolism and, at the same time, to increase lipid synthesis and glycolytic metabolism. Interestingly, loss of Rb causes abnormal expression of the farnesyl diphosphate synthase (FDPS), many prenyltransferases, and their upstream regulators SREBPs, in an E2F-dependent manner, leading to an increased isoprenylation and activation of N-Ras (89). Additionally, loss of Rb reduces the suppression of E2F (1 and 3), a mechanism that leads to promoter activation of prenyltransferase genes. Conversely, the presence of active Rb prevents the association of SREBPs with the FDPS promoter, suggesting that Rb negatively modulates the MVA pathway at both the transcriptional and the post-translational level.

## Cholesterol Contributes to Chemotherapy Resistance

Upregulated MVA pathway contributes to chemotherapy resistance by increasing both isoprenoids and cholesterol levels (10), thus generating a serious problem that arises in the treatment of many cancers. It has been shown that, in response to chemotherapy *in vitro*, some leukemic cells (i.e., AML cells) abnormally increased their cholesterol levels, whereas when this response is blocked with HMGCR inhibitors (i.e., statins), they increased its sensitivity to cytotoxic drugs (179). Interestingly, apoptosis resistance, typically observed in cancer (i.e., hepatocellular carcinoma (HCC), colon cancer and HeLa cells), has been related to cholesterol accumulation in mitochondria, resulting in decreased membrane fluidity (180–182). These data suggest that high mitochondrial cholesterol content contributes to chemotherapeutic resistance, especially to chemotherapeutic agents targeting mitochondria (182). On the other hand, as deeply discussed in a later section of the present review, it has been reported that HMGCR inhibitors (i.e., simvastatin) are able to overcome resistance or to potentiate the antitumoral effects of conventional chemotherapy in several models of cancer (*in vitro* and *in vivo*), such as, non-small cell lung cancer (183), resistant colorectal tumors (184) and human gastric cancer (39, 185). Therefore, inhibition of *de novo* cholesterol synthesis by statins may restore the efficacy and overcome resistance to conventional chemotherapy.

## Efficacy and Resistance to MVA Pathway Inhibitors in Human Tumors

**Figure 4:**
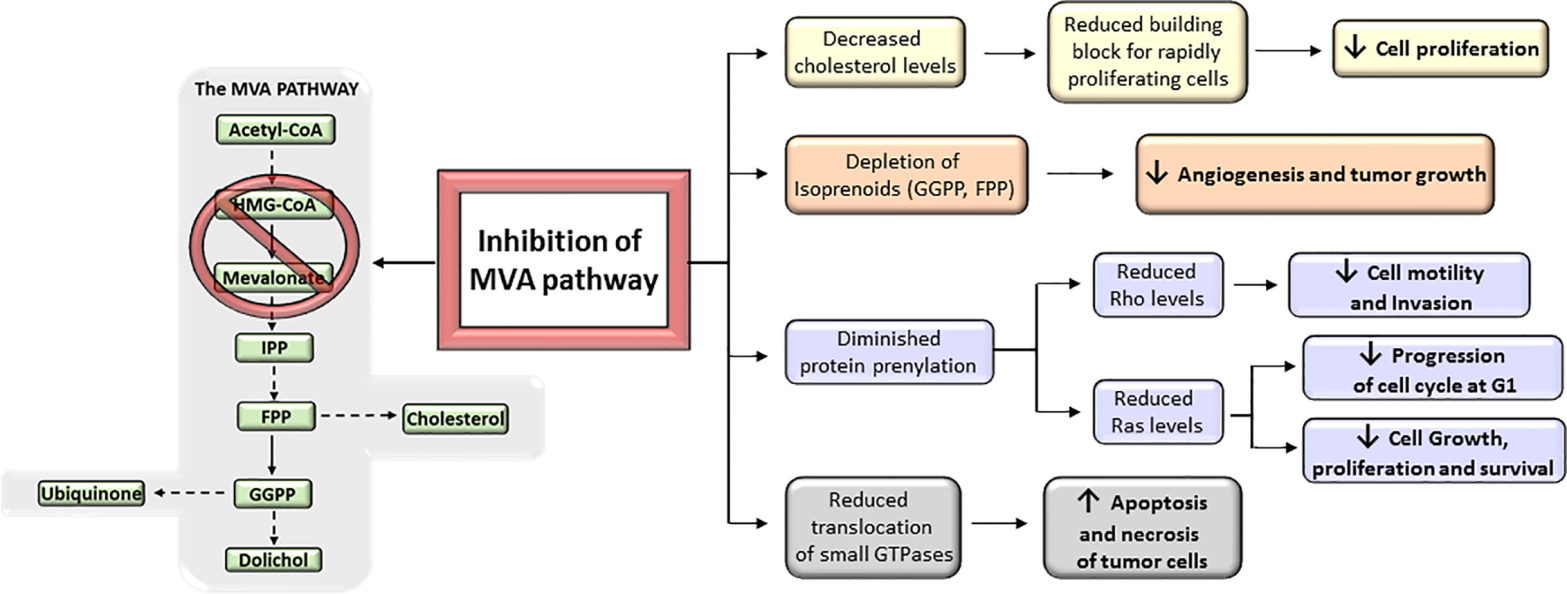
Antitumoral effects of mevalonate (MVA) pathway inhibition. The inhibition of MVA pathway triggers a series of anticancer events that get to inhibit tumor growth and progression. These include the reduction of MVA synthesis, which in turn decreases the levels of downstream products (isoprenoids) and therefore prevents protein prenylation; the reduction in the translocation of small GTPases such as Rho and Ras to the cell membrane; and the inhibition of cholesterol synthesis. All these inhibitory actions suggest that targeting the MVA pathway could represent a key mechanism to prevent cancer progression.

MVA biosynthetic pathway is considered a potential drug target to improve therapeutic window in cancer (11) (**Figure 4**). However, despite mounting body of preclinical and epidemiological evidences suggesting MVA pathway inhibitors (i.e., statins) as anticancer agents, many patients remained non-responsive to drug treatment in some cancer clinical trials (13, 20, 23, 30, 33, 186). This is, in part, because cancer cell selectivity, as well as predictive biomarkers of drug efficacy and drug resistance, is still poorly understood. Therefore, clinical trials are still required to further characterize the subset of cancers that are especially sensitive to MVA pathway inhibitors. The major limitation for the development of MVA pathway-based therapy is the absence of predictive biomarkers of efficacy and chemotherapy resistance, which is due to, at least in part, the lack of routine genotyping of human tumors. Therefore, predictive biomarkers, stratifications of patients, and selection of drug combination-based therapies may lead to a more effective MVA pathway-based therapy in cancer. Nowadays, there are a few completed clinical trials in which statins are used as monotherapy. Some of them have exhibited promising evidence of therapeutic potential and survival benefit mainly in breast cancer (187–189) and multiple myeloma (MM) (190, 191) (**Table 1**). Breast cancer clinical trials, using atorvastatin and fluvastatin, have shown decreased proliferation index marker in a subset of patients who were treated with cholesterol-management doses of statins between cancer diagnosis and surgery (187, 189) (**Table 1**). Moreover, a phase II window-of-opportunity trial has shown that high-dose atorvastatin (80 mg/day) induced anti-proliferative effects in breast cancer through cell cycle regulation *via* cyclin D1 and p27 (188) (**Table 1**). Although the molecular mechanisms are still unknown, hepatocarcinoma also seems to be particularly responsive to statins (193). Exposition to simvastatin has also been associated to reduced risk of hematological malignancies (194). Although clinical trials with statins show that some tumors may be more sensitive to statins than others, few of them have specifically enriched for subsets of patients whose tumors are preferentially sensitive to statins. As described above, tumors harboring an aberrant MVA pathway may be more sensitive to the antitumoral action of statins. This hypothesis follows the general principles of oncogene addiction and may provide the basis on which patients should be treated with statins. However, follow-up studies are still needed before validating those biomarkers to predict which cancers will be specifically sensitive to statin therapy. For example, certain phases of cancer progression, such as breast cancer recurrence, are particularly sensitive to the antitumoral effects of statins (195, 196). This is in line with the current paradigm of inter-patient cancer biodiversity. This lack of response might also be expected considering the evidence that the MVA pathway is regulated by many critical oncogenic signals. For example, a poor outcome has been reported in clinical breast cancer samples that carry a mutant form of p53 that stimulates the activity of MVA pathway (86). A molecular hallmark of basal transcriptome has been developed to forecast statin response in breast cancer *in vitro* (197) and aberrant MYC expression has been proposed as an indicator of statin response in specific cancer types (198). Notably, subsets of statin-sensitive and statin-insensitive cells were described in MM cell lines (199, 200). Remarkably, insensitive cells exhibited a robust feedback response, like normal cells, with an immediate up-regulation of different SREBP target genes, including HMGCR. In fact, recently, it has been reported that resistance of breast cancer cells to statins is, at least in part, due to the induction of HMGCR (201). Although the sterol feedback response tried to reinstate the MVA pathway, sensitive cells appear to show, in comparison with statin-insensitive cells, a weaker feedback response. This suggest that statin-sensitive cells have either lost checkpoint controls maintaining the MVA pathway intact, or that the pathway is deregulated and decreased HMGCR activity was not detected by the common intracellular sensors (i.e., SCAP, INSIGs). It appears that the sterol feedback response may serve as a protective mechanism, warranting that normal or statin-insensitive tumor cells are protected from the effect of statins. However, the loss of this sterol feedback response may not be a universal phenomenon across all statin-sensitive cancer types as it has been shown an intact sterol feedback response in AML cells (202). Moreover, tumor cholesterol may also be used a as a biomarker of statin sensitivity in many AML cells exposed to chemotherapy *in vitro* (202, 203). All these observations suggest that aberrant MVA pathway may be both a promoter of transformation and an indicator of statin sensitivity. Moreover, these data also establish the bases to further developing biomarker tools that could allow to predict which cancers are more sensitive to statins. That may provide a personalized medicine approach in which statins, and/or other inhibitors of MVA pathway, would constitute a successful class of anti-cancer drugs.

**Table 1:** Completed clinical trials with statins used in mono- or in combination therapy for cancer treatment.

Cancer type	Drugs	Research findings and conclusions	Study phase	References and ClinicalTrials.gov Identifier
Breast Cancer (BC)	Fluvastatin	Fluvastatin reduces tumor proliferation and increases apoptosis in high-grade, stage 0/1 BC. These data support further evaluation of statins as chemoprevention for ER-negative high-grade BC	Phase II	(187) NCT00416403
Breast Cancer (BC)	Atorvastatin	High-dose atorvastatin (HD-Atorv) induces anti-proliferative effects through up-regulation of tumor suppressor p27 and down-regulation of oncogene cyclin D1 in BC	Phase II	(188) NCT00816244
Breast Cancer (BC)	Atorvastatin	Atorvastatin and its metabolites are detectable in breast fine needle aspiration biopsies and its use is associated with decreased C-reactive protein (CRP). These results support atorvastatin further evaluation in phase II BC prevention studies	Phase I	(189) NCT100637481
Multiple Myeloma (MM)	Simvastatin	Standard-dose simvastatin (SD-Sim) is well tolerated without grade 3/4 toxicity and shows reduction of cell adhesion-mediated drug resistance in MM by inhibition of HMG-CoA-reductase. Moreover, authors suggest that SD-Sim efficacy needs to be improved either by dose escalation and/or by combination with other mevalonate pathway inhibitors	Phase II	(190) NCT00399867
Multiple Myeloma (MM)	Simvastatin	High-dose simvastatin (HD-Sim) has not beneficial effect on markers of bone turnover in MM. In fact, HD-Sim seems to be harmful rather than beneficial for MM patients due a transient stimulation of osteoclast activity	Phase II	(191) NCT00281476
Pancreatic Cancer (PC)	Simvastatin/Gemcitabine	Adding low-dose simvastatin (LD-Sim) to gemcitabine in treatment of advanced pancreatic cancer does not provide additional benefit but it also does not result in greater toxicity compared to gemcitabine alone. Since data point to an emerging role of statins in overcoming resistance to anti-epidermal growth factor receptor (EGFR) treatments, these results support further evaluation of efficacy and safety of combined LD-Sim and anti-EGFR agents (e.g., erlotinib or cetuximab) plus gemcitabine for treating advanced and metastatic PC	Phase II	(34) NCT00944463
Small-Cell Lung Cancer (SCLC)	Pravastatin/Etoposide/Cisplatin or Carboplatin	Pravastatin combined with standard platinum chemotherapy in patients with SCLC, although safe, does not benefit patients. Authors concluded that ongoing and future trials of statins used for either cancer prevention or treatment should monitor clinical efficacy and ensure that preclinical data are strong enough to warrant large-scale randomized studies	Phase III	(39) NCT00433498
Advanced Gastric Cancer (AGC)	Simvastatin/Capecitabine – Cisplatin (XP)	Addition of low-dose simvastatin (LD-Sim) to XP does not increase median progression free survival (PFS) in AGC, but it does not increase toxicity. Authors concluded that LD-Sim to chemotherapy is not recommended in untargeted patients with AGC. However, given the emerging role of statins as anti-cancer agents, this study also suggest that intermediate or high-dose simvastatin synergistically combined with standard chemotherapy regimens should be further evaluated in AGC	Phase III	(192) NCT01099085

## SREBP, a Drug Target to Increase Statin Efficacy and Overcome Drug Resistance

Inhibition of the MVA pathway leads to the activation of the SREBPs and the increased expression of MVA pathway genes, an effect that may be intensified in cancer cells and be responsible of statin resistance (201, 204). This SREBP-mediated feedback mechanism also increases the expression of the LDLR, and LDL-cholesterol uptake, which has been shown relevant in cancer cells (205, 206). Thus, the SREBPs work to replenish MVA-derived metabolites, which can depress the apoptotic response following statin treatment. Recent studies targeting the maturation or transcriptional activities of SREBPs supply proof of concept for the efficacy of SREBP inhibition in cancer therapy (204). Inhibiting the SREBP-regulated feedback response together with statin therapy could prevent drug resistance and increase the antitumoral efficacy of statins. In addition to HMGCR, the MVA pathway genes HMGCoAS1, GGPS1, SCAP, and SREBP2 are also good candidates to either suppressing other enzymes in the MVA pathway or blocking the SREBP-mediated feedback response in combination with statin therapy (207). Particularly, the clinically approved agent dipyridamole may be repurposed as an inhibitor of statin (fluvastatin)-induced SREBP processing and blocks the SREBP-regulated feedback response. This mechanism can potentiate antitumoral efficacy of statins, at least in prostate cancer, and most likely prevent drug resistance (208). However, preclinical and clinical investigations performed in order to investigate the utility of this combinatory drug strategy in cancer (i.e., HMGCR inhibitors *plus* SREBP inhibitors), are still a pharmacological challenge. Hopefully, other molecules can be repurposed as potentially antitumoral candidates in combination with statins. Thereby, fatostatin, a nonsterol diarylthiazole derivative originally developed to inhibit insulin-induced adipogenesis, suppresses (*in vitro* and *in vivo*) prostate cancer cell proliferation and induces apoptosis through inhibition of SREBP-regulated pathways, such as MVA pathway (209). Moreover, the combination of the anti-chronic myelogenous leukemia (CML) drug imatinib and simvastatin resulted in a synergistic killing effect on imatinib-resistant CML cells (210).

## Combinatory Therapy, a Strategy to Improve Therapeutic Windows of MVA Pathway Inhibitors in Cancer

Monotherapy with statins (e.g., simvastatin) displays anticancer activity *in vitro* (11). However, it is undefined whether lipophilic statins accumulate in tumor tissues at concentrations in which they are cytotoxic to cancer cells and efforts are still underway to determine tolerable and therapeutic dose of statins that could potentially be used in cancer (211). This is particularly jumbling as statins are also known to exert effects on certain normal cells. For example, myopathy is a rare but potentially dangerous side effect of statin treatment that is thought to be consequence of the induction of apoptosis in skeletal muscle cells (212). Interestingly, many studies have shown that statins can directly and specifically trigger the apoptosis of cancer cells (213, 214). Noteworthy, statins trigger apoptosis of cells derived from AML, whereas normal myeloid progenitors do not suffer apoptosis and keep a proliferative phenotype (213). This optimal therapeutic index may be result of the altered metabolic reprogramming of AML cells leading to an increased dependence on MVA-derived metabolites for survival and proliferation. These findings and the widespread use of statins for hypercholesterolemia control strongly suggest that these drugs might have a high therapeutic window to target tumors *in vivo*, despite the MVA pathway is active in both normal and cancer cells. Therefore, the therapeutic window of statins in cancer patients is being explored in several clinical trials that have been conducted to study the tolerability of high dose statins in cancer patients. Phase I–II clinical trials have shown that statins can be given to cancer patients in relatively high dosages (i.e., 15 mg/kg/day for simvastatin; 25 mg/kg/day for lovastatin). In these studies, the maximum tolerated dose of simvastatin was defined to be 15 mg/kg/day, 25-fold higher compared to a typical dose. However, response may not be satisfactory because to treat human cancer high doses of statins (10–100 μM) need to be used. Moreover, statins can cause anorexia and death in some individuals when serum concentrations reached levels higher than 20–25 μM (215, 216). An efficient strategy that might increase therapeutic window of statins in cancer patients is its combination with conventional chemotherapy in those cancers where altered aberrant cholesterol metabolism is linked to oncogenic signaling. This strategy can improve antitumor efficacy, by taking advantage of the synergistic effects of these drugs, and, potentially, reduces therapy-associated toxicity (8, 13, 217). For example and related to hematological cancers, cholesterol levels are abnormally elevated in many AML cells exposed to chemotherapy *in vitro* (202, 203). Suppressing this cholesterol response was further shown to sensitize AML cells to drug treatment, suggesting that MVA pathway inhibition by statins may improve the efficacy of conventional chemotherapy (203, 218). Thus, when pravastatin was combined with conventional treatment in AML resulted in complete or partial response in 60% of patients with AML (218). Furthermore, simvastatin also has potential application in oncohematology as it is able to potentiate the effects of imatinib in CML cells, inducing cell cycle arrest and apoptosis through the inactivation of STAT3 and STAT5 (210). Interestingly, lovastatin can enhance the antitumor effects of the antiretroviral drug saquinavir against human lymphoma cells, decreasing some of its side effects while potentiating the antitumor effectiveness (219). In another study, the combination of lovastatin with thalidomide and dexamethasone in patients with relapsed or refractory multiple myeloma prolonged overall survival and progression-free survival (220). Recently, it has been reported that combination of statins (atorvastatin, fluvastatin and simvastatin) and conventional chemotherapy (topotecan, paclitaxel and doxorubicin) acted synergistically to inhibit cell proliferation and to induce cytotoxicity in an aggressive natural killer cell leukemia (221). On the other hand, and related to solid tumors, a combinatory strategy has also been safely used to increase statin efficacy and security in HCC. Thus, pravastatin was combined with conventional treatment in HCC, resulting in significantly longer median survival (193). Moreover, promising results from both epidemiological studies (222, 223) and clinical trials (187, 224) suggest that patients with hormone dependent breast and prostate cancers, may benefit from the addition of statins to their conventional treatment regimens. Accordingly, it has been reported that simvastatin has additive effect with the antiandrogen enzalutamide promoting a greater inhibition of prostate cancer cells (225, 226). Moreover, simvastatin also enhances *ex vivo* the tumor cell inhibition effects of cisplatin or docetaxel in head and neck squamous carcinoma (HNSCC) (227) and sensitized human osteosarcoma cells to doxorubicin and cisplatin (228). Preclinical data have also shown that simvastatin in combination with cetuximab/irinotecan allows overcoming the resistance to irinotecan and oxaliplatin in KRAS mutant colorectal cancer (184). Moreover, simvastatin can potentiate the antitumor effect of capecitabine by suppressing proliferation and tumor invasion mediated by NFκB in a xenograft mouse model of human gastric cancer (185). In addition, it has been observed that lovastatin increases *in vitro* TNF-α -induced cell death in two gefitinib-resistant cholangiocarcinoma cell lines (229). Finally, statins can overcome the resistance to EGFR tyrosine kinase inhibitors in a non-small cell lung cancer cells (183) and to gefitinib in KRAS-mutant human non-small cell lung cancer cells (230). Paradoxically, several clinical trials have shown that combinatory therapy with statins does not add any benefit in comparison with conventional therapy. Clinical trials where simvastatin was combined with capecitabine–cisplatin (XP) in patients with previously untreated advanced gastric cancer (AGC) showed that addition of low dose (40 mg) of simvastatin to XP does not increased the median progression free survival (PFS) (192) (**Table 1**). Moreover, it has been reported that using a combination of pravastatin, a hydrophilic statin, with etoposide plus cisplatin or carboplatin in order to treat small-cell lung cancer does not provide additional benefit for patients (39) (**Table 1**). Alike, a randomized double-blind phase II clinical trial in which patients with locally advanced and metastatic pancreatic cancer participated, reported no clinical benefits when low doses of simvastatin were added to gemcitabine (34). The limited effectiveness of statins in these and previous studies (231, 232), and in clinical trials mentioned above (34, 39, 192) (**Table 1**) might be linked to low statin biodisponibility in cancer cells. Pharmacokinetic studies in chronic lymphocytic leukemia patients have shown that when simvastatin is administered at the maximum tolerated dose of 7.5 mg/kg, twice daily, plasma concentrations were dose proportional relative to the hypolipidemic doses, but lower than those required for *in vitro* cytotoxicity on cancer cells (231). This lower drug bioavailability in cancer cells might explain, at least in part, the absent or low efficacy of statins in cancer patients.

## Conclusions and Future Perspectives

Nowadays, an increasing amount of data, from preclinical and epidemiological studies, support an inverse association between the use of potent inhibitors of MVA pathway and the mortality rate in specific cancers (e.g., breast, colon, prostate, liver, hematological malignances). Furthermore, inhibitors of MVA pathway seem to have potential features that overcame main limitations of current chemotherapy: drug resistance and toxicity. Cancer treatment demands the use of relatively high doses of single inhibitors of MVA pathway for a prolonged period, thereby limiting this therapeutic strategy due to adverse effects. Clinically relevant, synergistic effects of tolerable doses of MVA inhibitors with conventional chemotherapy might enhance efficacy with lower doses of each drug and, probably, reduce adverse effects and resistance. In spite of that, clinical trials to identify combinatory therapies that improve therapeutic window are still a challenge. Dual strategies targeting both tumor cell proliferation and tumor niche and/or regulation of TIM represent a promising therapeutic strategy. However, despite TIM is known to be highly dependent of cholesterol biosynthesis, interplay of MVA biosynthetic pathway and TIM remains unexplored. Therefore, research needs to be performed in order to identify an effective MVA pathway inhibitor that may be clinically used, individually or in combination with conventional chemotherapy, in the treatment of cancers with addiction to cholesterol biosynthetic pathway.

## Author Contributions

All authors listed have made a substantial, direct, and intellectual contribution to the work, and approved it for publication.

## Funding

The Ministry of Science, Innovation and Universities (MCIU¸ SAF2015-65113-C2-2) with the funding of European Regional Development Fund-European Social Fund, SODECAN (Canary Islands Government; ET/15020), and FIISC (FUNCANIS2017; FIISC-DISA2018) supported research to Molecular and Translational Pharmacology Lab at University Institute for Biomedical and Health Research (IUIBS). Molecular and Translational Pharmacology Lab was also supported by grant-in-aid from Alfredo Martin-Reyes Foundation (Arehucas S.A.). MCIU-FPU16/00233 and ULPGC-2016 supported pre-doctoral programs to MG-R. and HA-T, respectively. The post-doctoral program Juan de la Cierva (MCIU2017; MCIU2019) supported to CR.

## Conflict of Interest

The authors declare that the research was conducted in the absence of any commercial or financial relationships that could be construed as a potential conflict of interest.
